# Measuring Mobile Phone Application Usability for Anticoagulation from the Perspective of Patients, Caregivers, and Healthcare Professionals

**DOI:** 10.3390/ijerph191610136

**Published:** 2022-08-16

**Authors:** Shih-Wei Wang, Chun-Chi Chiou, Chien-Hao Su, Cheng-Chih Wu, Shu-Chen Tsai, Tsu-Kung Lin, Chien-Ning Hsu

**Affiliations:** 1 Department of Pharmacy, Kaohsiung Chang Gung Memorial Hospital, Kaohsiung 833, Taiwan; 2 School of Pharmacy, Kaohsiung Medical University, Kaohsiung 807, Taiwan; 3 Department of Neurology, Kaohsiung Chang Gung Memorial Hospital and Chang Gung University College of Medicine, Kaohsiung 833, Taiwan; 4 Center for Mitochondrial Research and Medicine, Kaohsiung Chang Gung Memorial Hospital and Chang Gung University College of Medicine, Kaohsiung 833, Taiwan

**Keywords:** oral anticoagulants, mobile health technologies, eHealth, usability, health promotion, self-management, medication adherence, disease management, health professionals, patient

## Abstract

Oral anticoagulants (OAC) are recommended for preventing stroke and systemic embolism in atrial fibrillation. Proper use is imperative for maximizing anticoagulation therapy’s effectiveness and safety. In preparation for the implementation of a smartphone-based SmartMed app (application) aiming to promote patient self-management, medication adherence, and data collection for patients on anticoagulation therapy, its usability assessment can ensure the value of OAC app development and adoption. We evaluated the SmartMed app’s usability using the System Usability Scale (SUS) and the app-specific domain of the Mobile App Rating Scale (MARS) for its perceived impact on taking OAC regularly. We recruited 25 OAC users and their home caregivers and 59 healthcare professionals, including pharmacists, nurses, and cardiac surgeons from one medical center and one regional hospital in Taiwan. All participants (*n* = 84) thought the SmartMed app was useful, with mean SUS and MARS scores of 81.49 (±14.42) and 4.65 (±0.49), respectively. Usability evaluation revealed that fewer experiences with smartphone apps and different healthcare professionals (pharmacists versus nurses or cardiac surgeons) were associated with lower SUS scores and perceived impact. Throughout the evaluation process, the SmartMed app’s design was considered helpful from multiple stakeholders’ perspectives. Further ongoing mobile technology supports are necessary to establish the SmartMed app’s effectiveness.

## 1. Introduction

Cardiovascular diseases (CVD) are the leading cause of death globally. According to the World Health Organization (WHO), CVD statistics, approximately 17.9 million people died from CVDs in 2019 [1]. Similarly, heart attack and ischemic stroke ranked as the second (84.2/100,000 person) and fourth (51.6/10,000 person) leading causes of death in Taiwan, respectively [2]. Oral anticoagulants (OAC) are indicated for patients with atrial fibrillation (AF) and valvular heart diseases and for secondary prevention of ischemic stroke with cardioembolic etiology. Despite the availability of effective preventive medications, nonadherence to anticoagulants is associated with CVD morbidity, mortality, and health-related quality of life [3]. It is imperative to tailor individual patients’ preventive medicines to achieve the best effectiveness and acceptance. Underanticoagulation may cause a higher risk of thromboembolic events, while overanticoagulation is related to a greater risk of bleeding [4,5]. Moreover, both drug-drug and drug–food interactions may affect the therapeutic range of anticoagulation and cause potentially adverse events [6]. Therefore, it is necessary to educate patients taking anticoagulants regarding the medical information details and importance of proper adherence. Collecting information directly from patients can improve communication and prevent healthcare providers from underestimating the severity of the disease symptoms and adverse drug reactions.

Emerging mobile-based health applications (mHealth apps) via smartphones are widely used in patients with cancer [7,8,9], chronic kidney disease (CKD) [10], diabetes [11], chronic obstructive pulmonary disease (COPD) [12], etc. for education, symptom tracking, personal alerts and reminders, and so on and have been demonstrated to support traditional healthcare delivery and promote self-management. mHealth apps for AF demonstrated improved patient knowledge and behavior, medication adherence, and quality of life [13,14]. Existing mHealth apps supporting anticoagulant therapy for AF have largely focused on providing decision support to clinicians and early detection of AF to patients [15,16,17]. Limited smartphone apps have been developed focused on anticoagulants as a recent narrative review of 12 studies focused on warfarin [18]. It remains unclear what types of additional functionalities support, such as mHealth apps via smartphones, will be both effective and sustainable for healthcare delivery to facilitate patient self-management to optimize anticoagulant adherence and disease management. Additionally, some experts believe that too much information can cause confusion for the patient and interfere with medication adherence. It is also thought that communication barriers between healthcare providers and patients with low health literacy may influence self-management promotion for prevention and disease management [19]. Self-management experiences and patient attitudes and perceptions toward mHealth apps are very limited in Asian populations. Evidence is lacking on the mHealth apps perceived as useful in promoting patient self-management among healthcare professionals (HCP). The current study aimed to explore the usability of an evidence-based mobile smartphone app for anticoagulation (SmartMed app) from the patients, patients’ home caregivers, and HCP perspectives to establish an explicit understanding of the potential value added for patients in the future and support from HCP in obtaining value from mHealth apps.

## 2. Methods

### 2.1. Study Overview

The smartphone-based app and cloud-based system included six-tiered modules of the Smart Medication (SmartMed) app to better meet the care pathway for delivering tailored information to OACs users [4,5]. The contents of the SmartMed app were developed and maintained by a team of clinical pharmacists and physicians with specialties of cardiology and neurology from Kaohsiung Chang Gung Memorial Hospital (CGMH). The SmartMed app was initiated by the Department of Pharmacy, Kaohsiung CGMH and funded by the Ministry of Science and Technology in Taiwan.

The study participants were invited from the development site—Kaohsiung Chang Gung Memorial Hospital (CGMH), a medical center—and the regional hospital, Chia-Yi CGMH from the network of CGMH in Taiwan [20]. Kaohsiung CGMH was a tertiary medical center with 2720 beds, Chia-Yi CGMH was district hospital with 1351 beds, and both were located in Southwest Taiwan. A minimum number of at least 12 participants in both patient and/or home caregivers and HCP groups was applied in this usability study considering human mHealth app interaction [21].

**Figure 1:**
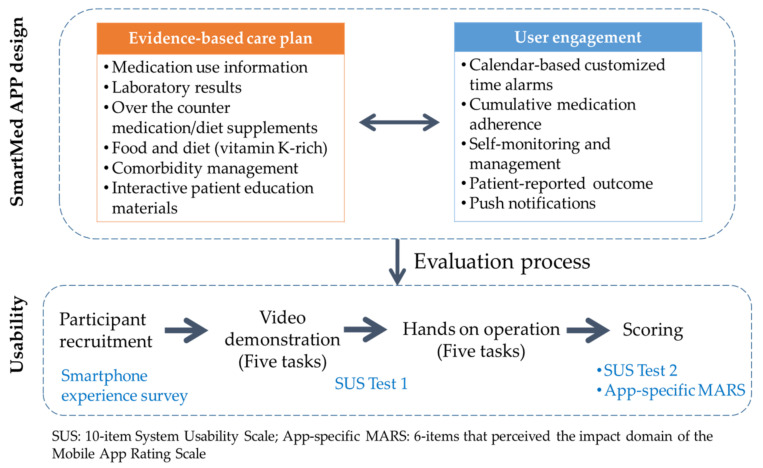
Study design and evaluation process.

Figure 1 shows the evaluation process. The usability of the SmartMed app was evaluated from the perspective of the patient, patient proxy (or home caregiver), and HCP using the 10-item System Usability Scale (SUS) [22] and a specific domain of the Mobile App Rating Scale (MARS) for the perceived impact of the app [23]. This study was conducted between April and September 2021.

### 2.2. Key Features of the Evaluation Program

**Figure 2:**
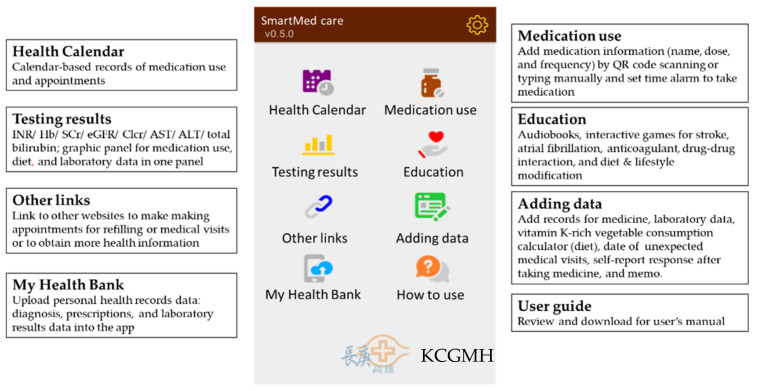
Main features of SmartMed app (KCGMH, Kaohsiung Chang Gung Memorial Hospital).

The SmartMed app was designed for OAC users (warfarin, dabigatran, rivaroxaban, apixaban, and edoxaban) to enhance patient medication adherence and engagement with obtaining self-management knowledge of medication use, laboratory, and special diet monitoring. In addition, the SmartMed app was expected to assist patients in better communicating with health professionals across different specialties and hospitals for tracking medication uses and management of disease symptoms. It included three-tier data components based on the current guidelines recommendation for anticoagulation therapy (i.e., medication use, testing results, and adding data), health calendar, education materials, additional links to making appointments (prescription refills, medical visits), and a link to upload electronic health information (Figure 2).

**Figure 3:**
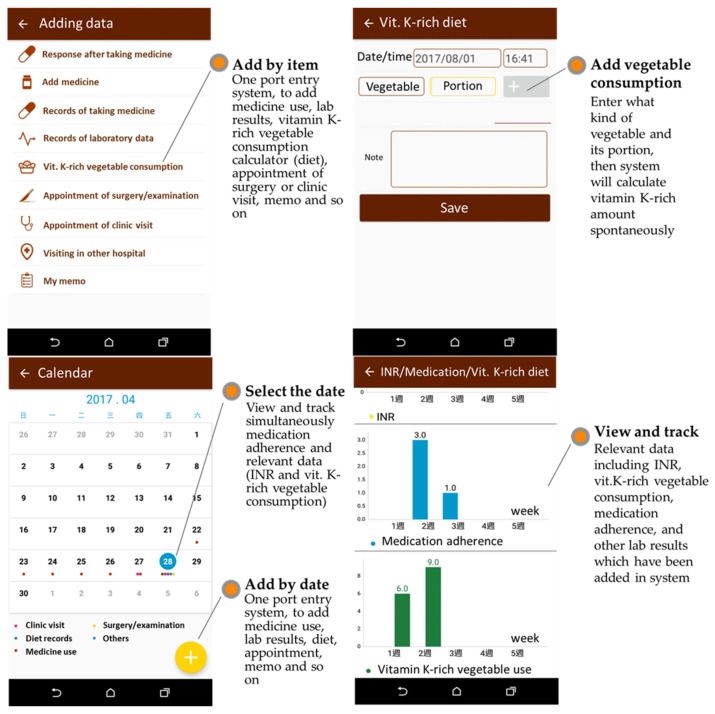
Example of the SmartMed App features.

To avoid information bias, study participants were requested to complete five prespecified tasks to experience and evaluate usability: (1) scan the prescription QR codes to add medication use information into the SmartMed app (domain of medication use) and schedule a reminder for taking medication (domain of health calendar); (2) input laboratory results, such as INR for warfarin users or eGFR for kidney function (domain of testing results); (3) calculate daily consumption of vitamin K-rich vegetables (domain of testing results) [24]; (4) experience the graphical panel, which encompassed medication uses, laboratory results (e.g., INR), and vitamin-K-rich vegetable consumptions for self-monitoring (Figure 3); and (5) patient education program as suggested by the global clinical guidelines for OAC treatment and management [4,5]. The patient’s education materials included an interactive game, audio, and an informative booklet for OAC.

### 2.3. Participant Recruitments

#### 2.3.1. Patient and Patient Proxy

Patients with chronic OAC therapy and their family caregivers were invited from an outpatient setting. Patient’s proxy (e.g., family caregivers) were recruited if the patient had a family caregiver to support their daily tasks, such as taking medications. A patient proxy was broadly defined as a family or nonfamily informal caregiver who assisted the older patient in healthcare-related activities in daily life. A well-trained assistant introduced the OACs app and followed the user manual in a participant-familiar language (e.g., local dialect) to help the focus group of patients and their family caregivers to gain a comprehensive understanding. Patients’ or their family caregivers’ perspectives were evaluated on a one-on-one basis.

#### 2.3.2. Healthcare Professionals

A study aim was to explore healthcare professionals (HCP) recommendation and support to enhance self-management skills of patients with chronic anticoagulation therapy—in particular, warfarin use. We invited pharmacists who were responsible for patient education, providing drug information, and the quality of pharmaceutical care monitoring. Post-graduate physicians, cardiovascular surgeons, and nurses were invited to evaluate the SmartMed app based on their experiences and primary duty of patient care with valve surgery and warfarin treatment. HCP participants installed the app on their mobile phones and used the app during the testing session. Their perspective was evaluated in a group training and evaluation process.

### 2.4. Evaluation Procedure

A self-reported smartphone use experience survey included sociodemographic data (age category, sex, and Android/iOS system of smartphone) and frequency of app use before the evaluation process (Figure 1). A trained pharmacist held a video demonstration to guide the participants’ first access on the five tasks on the OAC app. The app’s user manual was provided for patients, home caregivers, and HCP at the beginning of the evaluation process.

The first test of usability was performed at the end of video demonstration to provide a preview of the feasibility of the evaluation process. After the hands-on operation for the five prespecified tasks, the second test of usability and expected impact of the app on medication adherence were evaluated (Figure 1). The whole evaluation process per person was between 40 and 50 min.

### 2.5. Study Outcome

The usability measurement of the SUS, which contained 10 questions, was the most widely used standardized questionnaire for assessing perceived usability with multiple dimensions, which included efficiency, effectiveness, and satisfaction. Each item was measured on a 5-point Likert scale that ranged from 1 (strongly disagree) to 5 (strongly agree) (Table 1). The total SUS scores ranged from 0 to 100, and a higher score represented better usability [22].

The Mobile App Rating Scale (MARS) is a multidimensional, validated instrument for assessing the quality of mHealth apps [23,25]. The MARS includes five dimensions (engagement, functionality, aesthetics, information quality, and subjective) and one app-specific dimension to assess the perceived potential impact of the app on the user’s awareness, knowledge, attitudes, intentions to change, and actual change in the target health behavior [23]. The SmartMed app targeted specific patients treated with chronic disease for prevention or management and not for general wellness of health. Thus, only the app-specific section of the MARS (six items) was adopted, and the target behaviors for all the items were contextualized as “taking anticoagulant regularly” in our questionnaire. All items were rated on a 5-point scale that ranged from 1 (strongly disagree) to 5 (strongly agree).

### 2.6. Statistical Analyses

Participant’s data were deidentified. Data were presented as numbers and percentages, mean and standard deviation (SD), median and interquartile range (25th, 75th percentile). Differences among comparison groups were assessed using analysis of variance or t or chi-squared tests. A paired *t*-test was used to assess the difference between Test 1 and 2 SUS scores to assess the rater’s internal consistency. Both the SUS and app-specific domain of the MARS scores differences between the groups were analyzed using a generalized linear model. All statistical analyses were performed using SAS 4.0 (Cary, NC, USA). A two-sided *p*-value of <0.05 was considered statistically significant.

## 3. Results

### 3.1. Participant Characteristics

A total of 14 patients, 11 family caregivers, and 59 HCP completed the usability evaluation of the SmartMed app. The participants’ main characteristics are shown in Table 1. Of these, 75% of HCP were younger than 50 years, whereas more than half of the patients and caregivers were older than 50 (*p* = 0.0161). For the mobile app usage frequency, 92.86% patients reported ≥8 times per week (i.e., every day), followed by 72.73% family caregivers and 57.63% HCP. Most patients (60%) underwent warfarin therapy.

### 3.2. Usability of the SmartMed App

Table 2 presents the overall score and each item of the SUS. The mean total score was 74.14 (±13.27) at Test 1 and 82.63 ± 15.81 at Test 2.

The initial SUS score was higher in the patient group (81.49 ± 14.42) than other groups (HCP: 72.00 ± 14.10, caregivers: 76.36 ± 9.96). For Test 2, HCP rated a significantly higher score compared to Test 1 score (*p* < 0.0001). However, patients and their homecare caregivers ratings of Tests 1 and 2 of SUS remained consistent.

### 3.3. Perceived Impact of the SmartMed App

The results of the app-specific MARS score are presented in Table 3. The overall mean scores (full score = 6) were 4.40 and 4.68 in Test 1 and Test 2, respectively. Increased MARS score at Test 2 (compared to Test 1) was shown in the HCP and patient groups (HCPs: 4.34 vs. 4.71, *p* < 0.0001; patients: 4.62 vs. 4.71, *p* = 0.0401). The app demonstrated comparable scores for all domains of app-specific MARS score and was slightly superior regarding “knowledge” (item 2).

### 3.4. Factors Associated with Healthcare Professionals’ Evaluation of the SmartMed App

The associations between the characteristics of HCP and the SUS as well as the app-specific MARS score are displayed in Table 4.

Mean SUS score from pharmacists in CY-CGMH (non-medical center) were higher than pharmacists who worked in K-CGMH (medical center) (β = 12.88, 95% CI = 4.66–21.09, *p* = 0.0028). Both nurses and physicians gave lower SUS scores compared to pharmacists in K-CGMH (nurse: β = −30.02, 95% CI = −45.60–−14.44, *p* = 0.0003; physician: β = −20.26, 95% CI = −34.62–−5.90, *p* = 0.0067). App-specific MARS scores were comparable among pharmacists who worked in different hospitals (K- and CY-CGMH) (β = 0.1, 95% CI, −0.14–0.34, *p* = 0.4245). Compared to pharmacists in K-CGMH, slightly lower app-specific MARS scores were rated by nurses and physicians (nurse: β = −1.20, 95% CI = −1.66–−0.74, *p* < 0.0001; physician: β = −1.00, 95% CI = −1.42–−0.58, *p* < 0.0001).

Apart from different healthcare professionals (nurses and physicians versus pharmacists), lower-frequency smartphone apps users tended to be associated with lower SUS scores and perceived impact.

## 4. Discussion

### 4.1. Principle Findings

The current study reported that patients, patient proxies, and HCP had similar positive opinions regarding the OAC smartphone app regarding its usability and perceived effectiveness in helping patients take anticoagulant regularly. This is the first patient-centered mHealth app developed for promoting self-management, medication adherence, and data collection for patients on anticoagulation therapy with Mandarin language in Taiwan.

The current study yielded several factors that affected the level of usability of the SmartMed app. Limited experiences with smartphone apps and unfamiliarity with anticoagulation therapy may lead to underestimation of the usability of the SmartMed app. For instance, most participants rated low for “I need to learn a lot of things before I could get going with the app” and “I think that I would need the support of a technical person to be able to use this app,” which suggested that both patients and their homecare givers could use the app without difficulties. However, three caregivers reported seldom use (<4 times/week), whereas 92% of the patients used the app frequently (>8 times/week). This might explain why caregivers rated the app with lower SUS scores than the patients themselves (76.59 vs. 80.54). Among health professionals, SUS Test 2 scores were higher than those for Test 1, which suggests that the SmartMed app is easy to use and that hands-on operation experiences changed the health professional’s perceptions of app.

Although a moderate and positive correlation between age and usability of medical mobile device has been reported [26], user’s age was not associated with levels of usability in the present study participants. For instance, most HCP respondents were younger than 50 years, in contrast to both patient and caregiver groups; the mean scores of the SUS at Tests 1 and 2 were not different across participant groups. The usability between the patients and their caregivers in our study was comparable with the findings from previous studies in cancer and geriatric populations [27,28].

HCP-related factors associated with patient self-management promotion are not well known. Unlike pharmacists, who rated the OAC app with high usability, nurses and physicians had the opposite opinion. The SUS scores for non-pharmacist HCP (51.67 ± 1.44 for nurses and 63.13 ± 21.35 for physicians) were below the average SUS score of 68 [29,30]. The diverse findings and perspectives among patients and physicians were consistent with previous studies [31,32]. In our healthcare system, physicians are usually too busy to offer comprehensive medication information for patients in outpatient settings. The features and functionalities of the patient-centered OAC app were primary developed based on pharmacists’ experiences with patient counseling and medication review to identify drug–drug and drug–food interactions and dosing adjustments [33]. This may possibly explain the low usability score rated by non-pharmacist HCP.

The usability or value of mHealth techniques assessment was multidimensional. Six factors have been suggested to influence the value of mHealth intervention: the process of service delivery, organizational structure and professionals involved, cost of different treatments, burden of staff for delivering the intervention, lifestyle habits of the population, and local preferences on the quality of patient care [34]. The present study highlighted a need to develop consensus from different experts to empower patients’ self-management of health and medication uses (i.e., the attributes of health information) using the mHealth technology. Future mHealth-integrated care models should involve stakeholders, particularly multidisciplinary health professionals involved in the patient care process.

Despite the context and features, formative evaluations should be conducted to test design assumptions. Although the mHealth apps have the potential to save money through the improvement of treatment adherence and behavior changes, the cost-effectiveness of mHealth app remains unclear [35,36]. Despite the resource intensity in the development stage, the study team also realized a need for timely mobile technology support for the SmartMed app end-users after implementation to facilitate ongoing accessible and effective OAC care. From the developer’s perspective, ongoing cost was therefore necessary to improve health service delivery and engagement of patient and clinical outcomes through mobile communication novelties as well as the demand of security and privacy. Validation for evaluating usability with different language versions is necessary to promote the mHealth accessibility in different countries. The clinical value of different medication categories involves the indication for patients using the app of what the significant medicine-related features in daily care that must be added into the app are.

### 4.2. Strengths and Limitations

This was the first study on usability evaluation for OAC mHealth app from the perspectives of patients on anticoagulation therapy, family caregivers, and multidisciplinary HCP. The study design included various HCP, which helped us be more open regarding the development of an integrated care model to promote patient self-management and shared decision making. The pharmacists enrolled in the study included those from hospitals located in different geographic areas of Taiwan (rural versus non-rural cities). Pharmacists in non-rural cities gave higher SUS scores, which indicated that the OAC app could be used in different hospital levels.

There were some potential limitations of this study. First, the number of participants among HCP was proportionally different, which might limit the generalization of the findings in non-pharmacist HCP. Second, patients and their home caregivers who enrolled in this study were not new OAC users. Baseline knowledge and medication adherences of these respondents were not collected. Some respondents may be used to taking OAC regularly and be familiar with all health information related to their disease, medication, and diet, while some may not. The usability results may not be extrapolated to new users of anticoagulation. In contrast, the usability from these respondents may reflect what patient-related characteristics can benefit from the adoption of the mHealth app. Concerning that usability may be biased by end-users’ various response rates over a period of time; the short evaluation duration (<1 h) may not be sufficient to elucidate the true value of the usability of the SmartMed app among the study participants. Last, we provided an open-ended question in the scoring stage of this study (“Please give us your opinion or comment on this patient-oriented app”). Patients’ motivation to use mHealth app and strategies for user engagement with the mHealth app were reported by some nurses and pharmacists. Future investigations to identify factors associated with adoption and long-term engagement with the SmartMed app are warranted to investigate the promise of the mHeath app in daily care.

## 5. Conclusions

The patient-centered SmartMed app was considered usable and to have potential for maintaining medication adherence on anticoagulation therapy from different stakeholder perspectives, which included the end-users—patients and their home caregivers. Further research is warranted to establish the app’s effectiveness on improving self-management ability and prevention of thromboembolism and major bleeding in a real-world setting.

## Figures and Tables

**Table 1:** Characteristics of study participants.

	N of Patient	HCP (*n* = 59)		Patient (*n* = 14)		Family Caregiver (*n* = 11)		*p* Value *
Age group, year, *n*(%)								0.0161
≤35	19	18	(30.51)	0	(0.00)	1	(9.09)	
36–50	36	27	(45.76)	6	(42.86)	3	(27.27)	
51–65	24	13	(22.03)	6	(42.86)	5	(45.45)	
>65	5	1	(1.69)	2	(14.29)	2	(18.18)	
Sex, *n* (%)								0.1161
Male	39	24	(40.68)	10	(71.43)	5	(45.45)	
Female	45	35	(59.32)	4	(28.57)	6	(54.55)	
APP usage frequency/week, *n* (%)								0.2189
<1	11	9	(15.25)	0	(0.00)	2	(18.18)	
1–3	9	8	(13.56)	0	(0.00)	1	(9.09)	
4–8	9	8	(13.56)	1	(7.14)	0	(0.00)	
>8	55	34	(57.63)	13	(92.86)	8	(72.73)	
System of mobile phone, *n* (%)								0.1522
Android	51	32	(54.24)	10	(71.43)	9	(81.82)	
iOS	33	27	(45.76)	4	(28.57)	2	(18.18)	
Healthcare professionals, *n* (%)								-
Pharmacist in K-CGMH	36	36	(61.02)	-	-	-	-	
Pharmacist in CY-KCGMH	16	16	(27.12)	-	-	-	-	
Nurse	3	3	(5.08)	-	-	-	-	
Physician	4	4	(6.78)	-	-	-	-	
Patient’s age, year, mean (SD)		-	-	53.30	(10.29)	68.25	(24.73)	0.0831
OAC use, *n* (%)								0.1044
Warfarin	15	-	-	11	(78.57)	4	(36.36)	
Dabigatran	2	-	-	1	(7.14)	1	(9.09)	
Rivaroxaban	2	-	-	1	(7.14)	1	(9.09)	
Apixaban	3	-	-	1	(7.14)	2	(18.18)	
Edoxaban	3	-	-	0	(0.00)	3	(27.27)	
Drug Frequency, *n* (%)		-	-					0.6299
Once daily (QD)	5	-	-	2	(15.38)	3	(27.27)	
Twice daily (BID)	19	-	-	11	(84.62)	8	(72.73)	

* *p* value was based on chi-squared test with Fisher exact teat; OAC: oral anticoagulation; K: Kaohsiung (medical center); CY-CGMH: Chia-Yi Chang Gung Medical Hospital (regional hospital).

**Table 2:** Usability measure by SUS.

Item	Content	Overall (*n* = 84)					HCP (*n* = 59)					Patient (*n* = 14)					Family Caregiver (*n* = 11)				
		Test 1		Test 2		*p* Value	Test 1		Test 2		*p* Value	Test 1		Test 2		*p* Value	Test 1		Test 2		*p* Value
		Mean	(SD)	Mean	(SD)		Mean	(SD)	Mean	(SD)		Mean	(SD)	Mean	(SD)		Mean	(SD)	Mean	(SD)	
1	I think that I would like to use this product frequently	4.14	(0.75)	4.39	(0.68)	0.0004	4.02	(0.72)	4.44	(0.65)	<0.0001	4.43	(0.76)	4.36	(0.74)	0.3356	4.45	(0.69)	4.18	(0.75)	0.1921
2	I found the product unnecessarily complex	2.14	(0.95)	1.83	(0.98)	0.0021	2.27	(0.95)	1.75	(0.98)	<0.0001	1.57	(0.76)	1.86	(0.95)	0.3019	2.27	(0.90)	2.27	(1.01)	1.0000
3	I thought the product was easy to use	4.04	(0.80)	4.37	(0.77)	0.0002	3.92	(0.81)	4.36	(0.76)	0.0002	4.43	(0.76)	4.50	(0.76)	0.3356	4.18	(0.60)	4.27	(0.90)	0.7560
4	I think that I would need the support of a technical person to be able to use this product	2.36	(1.14)	2.05	(1.13)	0.0039	2.63	(1.13)	2.20	(1.19)	0.0014	1.57	(0.94)	1.43	(0.85)	0.4346	1.91	(0.70)	2.00	(0.89)	0.7961
5	I found the various functions in this product were well integrated	4.21	(0.71)	4.40	(0.75)	0.0041	4.15	(0.71)	4.41	(0.75)	0.0031	4.36	(0.74)	4.43	(0.85)	0.5830	4.36	(0.67)	4.36	(0.67)	1.0000
6	I thought there was too much inconsistency in this product	1.90	(0.77)	1.48	(0.69)	<0.0001	2.12	(0.74)	1.51	(0.73)	<0.0001	1.36	(0.50)	1.43	(0.65)	0.3356	1.55	(0.82)	1.36	(0.50)	0.1669
7	I imagine that most people would learn to use this product very quickly.	3.99	(0.83)	4.24	(0.80)	0.0074	3.80	(0.84)	4.22	(0.83)	0.0003	4.64	(0.50)	4.36	(0.93)	0.1039	4.18	(0.60)	4.18	(0.40)	1.0000
8	I found the product very awkward to use	1.82	(0.79)	1.46	(0.75)	<0.0001	1.92	(0.74)	1.51	(0.82)	0.0002	1.36	(0.84)	1.21	(0.43)	0.3356	1.91	(0.83)	1.55	(0.69)	0.1669
9	I felt very confident using the product	4.18	(0.75)	4.46	(0.70)	0.0001	4.05	(0.70)	4.44	(0.73)	<0.0001	4.79	(0.58)	4.71	(0.61)	0.3356	4.18	(0.87)	4.27	(0.65)	0.5884
10	I needed to learn a lot of things before I could get going with this product	2.01	(0.88)	1.75	(0.93)	0.0097	2.20	(0.88)	1.85	(1.01)	0.0155	1.29	(0.61)	1.36	(0.63)	0.3356	2.00	(0.77)	1.73	(0.65)	0.0816
Odd items (1, 3, 5, 7, 9)		15.56	(3.07)	16.87	(2.96)	<0.0001	14.93	(3.11)	16.86	(3.12)	<0.0001	17.64	(2.24)	17.36	(2.71)	0.4857	16.36	(2.38)	16.27	(2.45)	0.8461
Even items		14.76	(3.41)	16.43	(3.36)	<0.0001	13.87	(3.24)	16.19	(3.61)	<0.0001	17.86	(2.54)	17.71	(2.79)	0.7100	15.36	(2.98)	16.09	(2.30)	0.3328
Total score		74.14	(13.27)	81.49	(14.42)	<0.0001	72.00	(14.10)	82.63	(15.81)	<0.0001	81.07	(8.42)	80.54	(11.01)	0.7202	76.36	(9.96)	76.59	(9.17)	0.9187

**Table 3:** App-specific MARS for perceived impact.

Item	Content	Overall(*n* = 84)					Healthcare Professional(*n* = 59)					Patient (*n* = 14)					Family Caregiver (*n* = 11)				
		Test 1		Test 2		*p* Value	Test 1		Test 2		*p* Value	Test 1		Test 2		*p* Value	Test 1		Test 2		*p* Value
1	Awareness: This app is likely to increase awareness of the importance of [taking anticoagulant regularly]	4.45	(0.65)	4.68	(0.58)	<0.0001	4.38	(0.69)	4.68	(0.63)	0.0002	4.64	(0.63)	4.79	(0.43)	0.1648	4.45	(0.52)	4.55	(0.52)	0.3409
2	Knowledge: This app is likely to increase knowledge/understanding of [taking anticoagulant regularly]	4.43	(0.63)	4.74	(0.49)	<0.0001	4.40	(0.62)	4.76	(0.50)	<0.0001	4.57	(0.76)	4.86	(0.36)	0.1648	4.27	(0.65)	4.45	(0.52)	0.3409
3	Attitudes: This app is likely to change attitudes toward improving taking anticoagulant regularly	4.39	(0.64)	4.64	(0.65)	0.0010	4.33	(0.66)	4.68	(0.68)	0.0013	4.64	(0.63)	4.64	(0.63)	1.0000	4.27	(0.65)	4.45	(0.52)	0.3409
4	Intention to change: This app is likely to increase intentions/motivation to address [taking anticoagulant regularly]	4.42	(0.64)	4.68	(0.54)	<0.0001	4.33	(0.66)	4.73	(0.52)	<0.0001	4.71	(0.47)	4.64	(0.50)	0.5830	4.45	(0.69)	4.45	(0.69)	ND
5	Help seeking: Use of this app is likely to encourage further help seeking for [taking anticoagulant regularly] (if bleeding or thrombus symptoms occurred)	4.32	(0.68)	4.65	(0.55)	<0.0001	4.27	(0.71)	4.69	(0.53)	<0.0001	4.43	(0.65)	4.64	(0.50)	0.1894	4.36	(0.67)	4.45	(0.69)	0.3409
6	Behavior change: Use of this app is likely increase/decrease [taking anticoagulant regularly]	4.40	(0.60)	4.69	(0.54)	<0.0001	4.32	(0.62)	4.75	(0.51)	<0.0001	4.71	(0.47)	4.71	(0.47)	-	4.36	(0.67)	4.36	(0.67)	ND
mean score (SD)		4.40	(0.54)	4.68	(0.49)	<0.0001	4.34	(0.58)	4.71	(0.50)	<0.0001	4.62	(0.42)	4.71	(0.41)	0.0401	4.36	(0.53)	4.45	(0.49)	0.0816

ND: no difference; *p* value was based paired *t*-test.

**Table 4:** Factors associated with healthcare professional’s evaluation of the app.

		SUS					App-Specific MARS				
	*n*	Mean	(SD)	β	(95% CI)	*p* Value	Mean	(SD)	β	(95% CI)	*p* Value
HCP											
Pharmacist (K-CGMH)	36	81.81	(12.81)	Reference			4.80	(0.34)	Reference		
Pharmacist (CY-CGMH)	16	95.16	(5.44)	12.88	(4.66–21.09)	0.0028	4.94	(0.15)	0.10	(−0.14–0.34)	0.4245
Nurse	3	51.67	(1.44)	−30.02	(−45.60–−14.44)	0.0003	3.56	(0.51)	−1.20	(−1.66–−0.74)	<0.0001
Physician	4	63.13	(21.35)	−20.26	(−34.62–−5.90)	0.0067	3.96	(0.89)	−1.00	(−1.42–−0.58)	<0.0001
Age group, years											
≤35	18	86.67	(15.97)	Reference			4.89	(0.30)	Reference		
36–50	27	82.87	(15.04)	1.29	(−6.70–9.28)	0.7469	4.70	(0.43)	−0.06	(−0.29–0.18)	0.6329
>50	14	76.96	(16.47)	1.25	(−9.00–11.51)	0.8070	4.52	(0.75)	−0.05	(−0.35–0.25)	0.7402
Sex											
Male	24	82.40	(16.39)	1.06	(−6.62–8.74)	0.7823	4.73	(0.52)	0.06	(−0.16–0.29)	0.5673
Female	35	82.79	(15.63)	Reference			4.70	(0.50)	Reference		
Smartphone app uses/week, number of times											
<1	9	76.39	(20.62)	Reference			4.69	(0.52)	Reference		
1–3	8	87.81	(10.13)	2.75	(−10.52–16.03)	0.6784	4.85	(0.24)	−0.18	(−0.57–0.21)	0.3624
4–8	8	76.25	(16.48)	−1.23	(−14.29–11.83)	0.8506	4.38	(0.69)	−0.47	(−0.85–−0.09)	0.0173
>8	34	84.56	(15.03)	1.07	(−9.13–11.27)	0.8343	4.77	(0.48)	−0.20	(−0.50–0.10)	0.1840
System of mobile phone											
Android	32	83.83	(16.60)	Reference			4.71	(0.57)	Reference		
iOS	27	81.20	(15.01)	−4.57	(−11.74–2.60)	0.2063	4.72	(0.42)	−0.01	(−0.22–0.20)	0.8983

K (Kaohsiung)-, CY (Chia-Yi)-Chang Gung Memorial Hospital located in a rural city (medical center) and non-rural city (regional hospital), respectively.

## Data Availability

The data analyzed during this study are not permitted to be made publicly available.
